# Effect of Banxia Baizhu Tianma Tang for H-type hypertension: A protocol for a systematic review

**DOI:** 10.1097/MD.0000000000019309

**Published:** 2020-02-28

**Authors:** Dongjie Chen, Chenyue Li, Hairong Cai, Jieqin Zhuang, Yonglian Huang, Xiaohong Peng, Shaoping Li, Yaxiu Huang, Ping Wang, Yajie Luo, Zhenye Zhan

**Affiliations:** a Department of Critical Care Medicine, Beijing University of Chinese Medicine Shenzhen Hospital; b The Basic Medicine College of Guangzhou University of Chinese Medicine; c The Second Clinical Medical School, Guangzhou University of Chinese Medicine; d Department of Emergency, Panyu District Central Hospital of Guangzhou, Guangzhou, Guangdong Province, China.

**Keywords:** Banxia baizhu tianma tang, H-type hypertension, protocol, systematic review

## Abstract

**Background:**

H-type hypertension is a kind of cardiovascular disease that threatens human life and health seriously. Banxia Baizhu Tianma Tang (BBTT) has been used widely for H-type hypertension while its effective evidence is not clear. Hence, we provide a systematic review protocol to evaluate the efficacy and safety of BBTT in the treatment of H-type hypertension.

**Methods:**

Nine databases including Cochrane Library, PubMed, EMBASE, WOS, Medline, CNKI, WangFang, CBM, and VIP will be searched from their inception to October 2019. All randomized controlled trials (RCTs) of BBTT for H-type hypertension will be included. The language is limited to Chinese and English. The primary outcome measure will be the major adverse cardiac and cerebral events (MACCE). The entire process will include study selection, data extraction, assessment of bias risk, data synthesis. Data analysis will be performed using RevMan V.5.3.5 (The Cochrane Collaboration, Oxford, UK).

**Results:**

This study will evaluate the efficacy and safety of BBTT in the treatment of H-type hypertension from several aspects, including MACCE, blood pressure (BP), blood lipids, inflammation indicators and homocysteine (Hcy).

**Conclusion:**

This systematic review will provide evidence for determining whether or not BBTT is an effective and safe intervention for H-type hypertension.

**PROSPERO registration number:**

PROSPERO CRD 42019131491.

## Abstract 2

Supplemental Digital Content is available in the text

## 1 Introduction

H-type hypertension is primary hypertension with elevated plasma homocysteine (Hcy) (plasma Hcy level >10 μmol/L).^[1]^ High-cysteine, low-folate,^[2–4]^ and methylenetetrahydrofolate reductase (MTMFR) gene 677TT genotypes^[5]^ are common in Chinese patients with hypertension. Epidemiological investigation shows that the proportion of H-type hypertension in hypertension patients in China is as high as 75.0% to 80.3%.^[6,7]^ China's third national cause-of-death survey report shows that cerebrovascular disease has become the number one cause of death in our population, and the incidence of stroke is increasing at a rate of 8.7% per year.^[8]^ Studies have confirmed that both hypertension and hyper-Hcyemia are important risk factors for stroke.^[9–13]^ Hcy is a cytotoxic sulfur-containing amino acid, which is an intermediate metabolite produced by methionine demethylation.^[14]^ High Hcy levels are closely related to cardiovascular and cerebrovascular diseases, peripheral vascular diseases, and diabetes. High Hcy levels and hypertension have synergistic effects in the process leading to cardiovascular and cerebrovascular diseases.^[15,16]^ Sacco et al^[17]^ found that the incidence of stroke in patients with hyper-Hcyemia was higher than that in patients with normal Hcy, and that of those with hypertension also had a higher incidence of stroke than those with normal blood pressure and Hcy.

At present, the conventional treatment of coronary heart disease by western medicine mainly includes drug therapy, however, it could result in certain side effects and poor compliance, which can no longer meet the needs of comprehensive management of H-type hypertension.

Traditional Chinese medicine (TCM) is an important part of complementary and alternative medicine (CAM), which has been widely accepted in China and applied in practice.^[18]^ Banxia Baizhu Tianma Tang (BBTT) is composed of 6 kinds of TCM: Banxia (*Pinellia tuber*), Tianma (*Gastrodia elata*), Fuling (Indian bread), Juhong (Citrusmaxima), Baizhu (*Atractylodes macrocephala*), Gancao (Liquorice root), all of which are standardly marked in Chinese Pharmacopoeia (V.2015). BBTT has been often used in the treatment of H-type hypertension in clinical practice in China with uncertain effects.^[19,20]^ However, to our knowledge, there is no systematic review of its efficacy and safety in the treatment of H-type hypertension. Therefore, we propose the current protocol to evaluate the effectiveness and safety of BBTT on H-type hypertension, providing a reference for clinical use.

## 2 Methods

### 2.1 Inclusion criteria for study selection

#### 2.1.1 Types of studies

This systematic review will be included in all randomized controlled trials (RCTs), excluding any non-randomized controlled trials such as controlled clinical trials, case series, case reports, animal experiments. And repeated publications, data rereading literature, literature with incomplete indicators will also be excluded. Considering the language limitations of our researchers, the literature included will be limited to English or Chinese.

#### 2.1.2 Types of patients

We will include patients with H-type hypertension. There is no limit to sex, ethnicity, education, economic status, disease severity.

#### 2.1.3 Types of interventions

The control group was treated with conventional treatment, including folic acid tablets and antihypertensives, and combined treatment of BBTT and conventional treatment was used in the experimental group.

We will include various dosage forms of BBTT, including tablets, capsules, pills, powders, and extracts. We will exclude RCTs in which BBTT is combined with other Chinese medicine methods, such as acupuncture and moxibustion. There is no limit to dose and route of administration.

#### 2.1.4 Types of outcome measures

##### 2.1.4.1 Primary outcomes

The primary outcomes will be major adverse cardiac and cerebral events (MACCE), including nonfatal myocardial infarction, cerebral ischemic stroke (CIS), cerebral hemorrhagic stroke (CHS), coronary revascularization, coronary heart disease death.

##### 2.1.4.2 Secondary outcomes

Systolic blood pressure (SBP), diastolic blood pressure (DBP), high sensitive C reaction protein (hs-CRP), Interleukin-6 (IL-6), matrix metalloproteinases-9 (MMP-9), blood lipids, Hcy, adverse drug reactions.

### 2.2 Search methods for the identification of studies

Nine databases including Cochrane Library, PubMed, EMBASE, WOS, Medline, CNKI, WangFang, CBM, and VIP will be searched from their inception to October 2019. English search terms include: Banxia Baizhu Tianma Decoction, H-type Hypertension, and RCTs. The strategy for searching the PubMed will be shown as an example in Appendix A (Supplemental Appendix A), and modified by using other databases.

#### 2.2.1 Searching other resources

Meanwhile, we will search for references, conference papers, and dissertations included in the study manually. In addition, we will search the WHO International Clinical Trial Registry Platform (ICTRP), Baidu Academic and Google Academic.

### 2.3 Data collection and analysis

#### 2.3.1 Selection of studies

**Figure 1:**
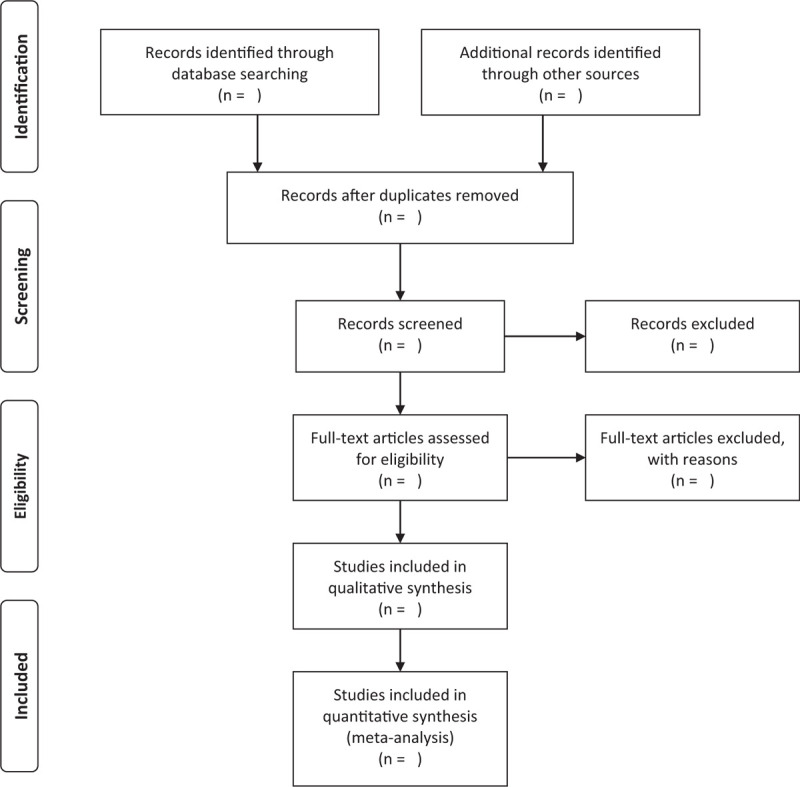
Preferred Reporting Items for Systematic review and Meta-Analysis (PRISMA) flow chart.

First, 2 authors will exclude the obvious disqualified literatures or duplication independently by screening the titles and abstracts. Secondly, they will assess the full-text of the studies and confirm the eligibility for the review. If there are any disagreements, the problems will be resolved by discussion or consulting the third author. The process of studies selection and meta-analysis is presented in an adapted Preferred Reporting Items for Systematic review and Meta-Analysis (PRISMA) flow diagram (Fig. 1).

#### 2.3.2 Data extraction and management

Two researchers will screen the literature and extract the data according to the pre-designed extraction table, including the author, publication time, research objects, intervention measures, and outcome indicators independently.

In cases where agreement cannot be reached, the matter will be settled after consulting experts and arbiter.

#### 2.3.3 Assessment of risk of bias in included studies

The assessment of risk of bias the included literature will be performed according to the “risk of bias” tool recommended by the Cochrane Handbook V.5.3, including random sequence generation, allocation concealment, blinding of participants, personnel and outcome, incomplete outcome data addressed, selective reporting, and other bias. Ultimately, the risk of bias will be categorized into 3 levels: low, unclear, or high in accordance with the quality classification criteria. The 2 reviewers will evaluate the quality of the literature independently. If the opinions were not uniform, they will resolve it through discussion or consulting the third researcher.

#### 2.3.4 Measures of treatment effect

For continuous data, the result will be presented as the weighted mean difference (WMD) or the standardized mean difference (SMD) with 95% confidence interval (95% CI). As for discontinuous data, the result will be presented as the relative risk (RR) or odds ratio (OR) with its 95% CI.

#### 2.3.5 Dealing with missing data

We will try to get missing data by contact the corresponding author. If that fails, the analysis will be conducted based on the available data and the potential impact will be analyzed during the discussion.

#### 2.3.6 Assessment of heterogeneity

First, the heterogeneity will be evaluated by *I*^2^ statistic and chi-squared test. *I*^2^ <50% will be considered as no statistical heterogeneity, while *I*^2^ ≥50% will be taken as high statistical heterogeneity. In cases of high heterogeneity, further subgroup analysis or sensitivity analysis should be conducted to find the source of heterogeneity.

#### 2.3.7 Assessment of reporting bias

If the review include enough literature (≧10 trials), we will use the Egger's funnel plot to evaluate publication bias.

#### 2.3.8 Data synthesis

We will perform data synthesis by using RevMan5.3 software. Heterogeneity tests were performed for each study. If there is no statistic heterogeneity, the fixed effects model will be used to conduct meta-analysis. If there is heterogeneity between studies, the source of heterogeneity should be analyzed first. The random effects model will be performed for meta-analysis when clinical heterogeneity is small. Descriptive analysis will be performed if the heterogeneity is too large or the source of heterogeneity is unknown. If the sample size is ≤1, only general statistical descriptions will be made.

#### 2.3.9 Subgroup analysis

Subgroup analysis will be performed to explore heterogeneity based on sex, age, courses, disease condition, race, kinds of BBTT.

#### 2.3.10 Sensitivity analysis

We will perform sensitivity analysis based on sample size, impact of data loss, and methodological quality if there are sufficient data available.

#### 2.3.11 Grading the quality of evidence

The quality of evidence will be evaluated using the Grading of Recommendations Assessment, Development and Evaluation (Version 3.6, The GRADE Working Group, 2010). The quality of evidence was divided into 4 levels: high, medium, low, and extremely low.

## 3 Discussion

With the aging of the population and the acceleration of urbanization, H-type hypertension has become a serious public health problem, which seriously affects human life and health. Drugs are the most important treatments for treating H-type hypertension. However, there are certain side effects about these treatments. BBTT may be a useful treatment for H-type hypertension, and it is unlikely to produce severe side effects. As far as we know, it is unclear whether BBTT is effective and safe intervention for H-type hypertension. Therefore, we aim at providing evidence to clinicians so that more and more patients with H-type hypertension may also benefit from alternative interventions. However, there are some certain potential limitations in this systematic review. First, the language is limited to Chinese or English, which may result in selection bias. Second, different dosage of herbs, the age of the patient, and the severity of H-type hypertension may present a heterogeneity risk. Finally, small samples of RCTs may lead to high risks of bias.

## Author contributions

**Conceptualization:** Yaxiu Huang.

**Data curation:** Zhenye Zhan.

**Funding acquisition:** Ping Wang.

**Investigation:** Chenyue Li, Jieqin Zhuang.

**Methodology:** Yonglian Huang.

**Project administration:** Ping Wang.

**Software:** Xiaohong Peng.

**Supervision:** Ping Wang, Yajie Luo, Zhenye Zhan.

**Validation:** Shaoping Li.

**Writing – original draft:** Dongjie Chen, Jieqin Zhuang, Chenyue Li, Hairong Cai.

**Writing – review & editing:** Dongjie Chen, Jieqin Zhuang, Hairong Cai, Chenyue Li.

**Dongjie Chen orcid:** 0000-0002-2399-2431.

**Chenyue Li orcid:** 0000-0002-6800-4388.

**Hairong Cai orcid:** 0000-0002-0332-2035.

**Jieqin Zhuang orcid:** 0000-0001-6331-163X.

**Yonglian Huang orcid:** 0000-0002-2453-7215.

**Xiaohong Peng orcid:** 0000-0002-3452-5441.

**Shaoping Liorcid:** 0000-0002-3514-2756.

**Yaxiu Huang orcid:** 0000-0001-7448-1462.

**Ping Wang orcid:** 0000-0002-6342-1413.

**Yajie Luo orcid:** 0000-0002-2050-3268.

**Zhenye Zhan orcid:** 0000-0002-7108-3283.

## Supplementary Material

Supplemental Digital Content
