# Supplementary material for: Effect of Banxia Baizhu Tianma Tang for H-type hypertension: A protocol for a systematic review
Source: Medicine (Baltimore). 2020 Feb 28;99(9):e19309. doi: 10.1097/MD.0000000000019309 (PMC7478445; doi:10.1097/MD.0000000000019309)
Supplement: Supplemental Digital Content [file medi-99-e19309-s001.doc]

**Appendix A.**

***Search strategy used in PubMed database***

#1 H-type Hypertension

#2 Banxia Baizhu Tianma Decoction OR Ban xia Bai zhu Tian ma decoction OR Banxia Baizhu Tianma tang OR Banxia Baizhu Tianma yin

#3 Randomized controlled trial OR clinical study OR Clin-ical Trial OR Controlled study OR Controlled Trial OR Random*Control* study OR random* Control* Trial

#1 AND #2 AND #3
